# Application of Artificial Intelligence in Medicine: An Overview

**DOI:** 10.1007/s11596-021-2474-3

**Published:** 2021-12-06

**Authors:** Peng-ran Liu, Lin Lu, Jia-yao Zhang, Tong-tong Huo, Song-xiang Liu, Zhe-wei Ye

**Affiliations:** Department of Orthopedics, Union Hospital, Tongji Medical College, Huazhong University of Science and Technology, Wuhan, 430022 China

**Keywords:** artificial intelligence, medicine, application, overview

## Abstract

Artificial intelligence (AI) is a new technical discipline that uses computer technology to research and develop the theory, method, technique, and application system for the simulation, extension, and expansion of human intelligence. With the assistance of new AI technology, the traditional medical environment has changed a lot. For example, a patient’s diagnosis based on radiological, pathological, endoscopic, ultrasonographic, and biochemical examinations has been effectively promoted with a higher accuracy and a lower human workload. The medical treatments during the perioperative period, including the preoperative preparation, surgical period, and postoperative recovery period, have been significantly enhanced with better surgical effects. In addition, AI technology has also played a crucial role in medical drug production, medical management, and medical education, taking them into a new direction. The purpose of this review is to introduce the application of AI in medicine and to provide an outlook of future trends.

Artificial intelligence (AI) is a new technical discipline that uses computer technology to research and develop the theory, method, technique, and application system for the simulation, extension, and expansion of human intelligence. The conception of AI first appeared in 1950 from the scientist Alan Turing, who is named as the "father of artificial intelligence"; he developed the "Turing test" and described AI as similar to but more complex than the human brain[1, 2].

With the development of AI in recent years, especially the appearance of deep learning (a branch of computer learning algorithms and the core composition of a new generation of AI technology, which can automatically learn from big-data-analysis and then artificially and independently make decisions upon the knowledge, including various neural networks such as the deep belief network, convolutional neural network, long- and short-term memory network, etc.), there has been a surge of interest regarding this new technical discipline and the promotion of a series of artificial intelligent systems in practical applications, like the Internist-1 system[3], MYCIN system[4], CASNET system[5], and also some databases and record systems[6]. Now AI is widely applied to multiple fields and plays a significant role in technical improvements, thus leading to a new conception: Artificial Intelligence plus (AI plus). AI plus takes advantage of the achievements and technology of AI and combines them with traditional industries to create new productivity, innovation, and development. AI research has demonstrated that the output-input ratio in medicine is more promising than that of other fields[7]. The combination of AI and medicine (AI plus medicine) changes the traditional medical model and makes a revolutionary promotion. AI plus medicine has also attracted much attention due to its potential prospects and future. Therefore, this paper aims to review the latest applications of AI plus medicine in recent years.

## 1 AI IN MEDICAL DIAGNOSIS

When a clinician diagnoses a patient with a certain illness/condition with the assistance of AI, the time required for a diagnosis can be greatly reduced and the diagnostic efficiency can be significantly improved. By analyzing the clinical data from radiology (like X-ray, CT, and MRI), pathological, endoscopic, ultrasonographic, and biochemical examinations for related human body indicators, AI can output results quickly and change the ineffective traditional medical model, which is unable to give timely and accurate conclusions, especially for complicated diagnoses. In addition, as AI can solve problems in such a short time, doctors can make a more deliberate and reasonable treatment plan according to the patient's condition.

Peng-ran LIU, E-mail: lprlprlprwd@163.com; Lin LU, E-mail: lledu2014@163.com
[#]Corresponding author, E-mail: yezhewei@hust.edu.cn
[†]The authors contributed equally to this work.
[*]This project was supported by the National Natural Science Foundation of China (No. 81974355).

### 1.1 AI in Radiology

As a scientific and intuitive basis for medical diagnosis, radiology is presently involved in the diagnostic process of almost all diseases. The demand for a radiological diagnosis is increasing at a high rate per year; however, the cultivation of medical talent cannot be achieved overnight and the number of medical doctors with radiation medicine experience is increasing slowly. As the gap between the supply and demand of medical doctors in this field is constantly increasing, high occupation pressure and misdiagnosis rates are observed. Therefore, it is of great practical significance to find other ways such as AI to temper the urgent situation. In radiological diagnosis, numerous applications of AI have appeared in recent years. For example, during AI deep learning research, Francesco found a new algorithm with a high sensitivity for the early screening, rapid diagnosis, and grading of retinal diseases[8], which also has been confirmed in other research[9, 10]. In addition, Gong[11] invented an AI-aided diagnostic (CADx) system based on clinical CT data to classify malignant and benign lung nodules by analyzing 243 patients with confirmed pulmonary nodules; he demonstrated the feasibility of the CADx system for accurately distinguishing the nature of lung nodules and the practicability of the system for early and subtle lung cancer diagnosis. Moreover, his team combined the quantitative imaging (QI) features (AI technology) and serum biomarkers of 173 patients with pulmonary nodules with the system to improve its performance of pulmonary nodule classification and finally got an optimistic result, which demonstrated a better CADx system performance by using QI features than only using the serum biomarkers[12]. There is also a study for the effectiveness between clinical doctors and AI by Rodriguez-Ruiz[13], who compared the stand-alone performance of an AI system to that of radiologists in breast cancer diagnosis by X-ray; based on 2652 exams overall, the performance of the AI system was statistically not inferior to that of clinical radiologists, meaning that the AI system in this study reached an accuracy equal to that of clinical radiologists with expertise in breast cancer diagnosis. Similarly, breast cancer screening augmented with intelligent technology defeated clinical radiologists, showing a lower misdiagnosis rate and a workload decrease of 88%[14]; additionally, the automatic preselect function significantly reduced the pressures on clinicians[15]. Furthermore, in MRI data analysis, an AI algorithm also has been shown to be superior to that of human observers, immensely improving the positive rate of subtle inflammatory detection in early rheumatoid arthritis[16].

### 1.2 AI in Pathology

Pathology is the footstone of the diagnosis of tumors and other lesions. With the development of pathological scanning techniques and the upgrade of related software, whole-slide imaging technology has become a routine diagnostic method in pathological work. However, there are still problems about how to automatically and rapidly analyze and obtain an accurate diagnosis from the convenient pathological images, thus demanding a prompt solution. Some scholars have pointed out that “AI is the next step and future of precision pathology” and have proposed a new blueprint[17–21]. As a means to predict a diagnosis, the application of AI in pathology has shown bright prospects. During the analytical work, with augmentation of the AI algorithm, the processes of pathological image segmentation, tumor identification, and metastasis determination have been promoted, and the work is finished in a higher quality and a shorter period[22]. In addition, research has shown that even in some cases, the AI algorithm outperforms professional clinical pathologists for diagnosis based on pathological images[23]. For example, Hart has utilized the convolutional neural network to distinguish Spitz and conventional melanocytic lesions, two different pathological types, and the results show an extremely high accuracy[24]. Kosaraju also has proposed an innovative multi-task model using AI deep learning that can simultaneously take multi-scale patch images for pathological image analysis. After assessing the pathological images of well-, moderately, and poorly differentiated gastric cancer, the efficient and accurate performance of the new model outperformed other contemporary AI methods[25]. Moreover, Coudray confirmed that the deep learning model could predict six genetic mutations associated with cancer and assist doctors to detect the subtype and gene mutation in cancer diagnosis, with a high accuracy of 97%; the model code is published online[26]. Furthermore, AI has been used in the diagnosis of epithelial tumors, lung cancer, basal cell carcinoma, and glomerulosclerosis[27–31]. These developments emphasize the practicability of AI technology applied in pathology.

### 1.3 AI in Endoscopy

As for AI in endoscopy, with AI technology augmentation, there have been great advances in endoscopy detection that have changed the traditional model and improved the efficiency. According to some experts, AI technology can effectively promote the detection of lesions, colorectal polyps, as well as gastric and esophageal cancer using endoscopy[32]. Gulat and Emmanuel have pointed out that endoscopy is an attractive technology for AI augmentation with immense potential; after deep learning, the AI system can significantly enhance the diagnosis of stomach and intestinal diseases, including Barrett’s esophagus, squamous carcinoma, and gastric cancer, by shortening the detection time and improving the diagnostic accuracy[33]. In addition, some scholars have collected

7556 clinical images by endoscopy and then analyzed them by AI technology to provide a practical neural network algorithm to automatically detect bowel lesions; the results showed that endoscopy combined with the new AI algorithm had a higher sensitivity and more accurate localization of the bowel lesions than with the traditional model[34]. With more and more research confirming the feasibility of AI plus endoscopy in the diagnosis and classification of various diseases[35–37], there is obviously a future for this new technology.

### 1.4 AI in Ultrasonography and Biochemical Examinations

Similar to above, the application of AI technology also raises the level of diagnosis using ultrasonography and biochemical tests. Although image-based computer-aided diagnosis (CAD) systems have already been applied by doctors to diagnosis through ultrasound, the performance is largely dependent on the detection and classification methods. Combined with AI technology, methods have changed a lot. For example, Nguyen has proposed a new ultrasonographic image analysis method based on AI that successfully enhanced the consequence of thyroid nodule classification[38]. Other scholars have also confirmed that the use of AI can promote the traditional ultrasonographic detection of tumors in the thyroid, breast, bronchia, puborectalis muscle, and urogenital hiatus as well as other obstetric and gynecological lesions, with a high efficiency and accuracy[39–44].

What's more, after a comprehensive learning analysis of clinical examination data, based on the model of big-data analysis, AI has also made significant progress in clinical disease diagnosis. By utilizing deep sequencing to analyze genes that are recurrently mutated in acute myeloid leukemia (AML) and large electronic health record database analysis, Abelson has developed an AML predictive system contributing to earlier detection and monitoring[45]. In addition, Sun successfully predicted the radiomic signature and clinical outcomes in anti-PD-1/PD-L1 immunotherapy by analyzing the CT images and RNA sequencing data from patients with malignant tumors using an AI algorithm[46]. By analyzing the gene and related clinical features, AI also facilitates the diagnosis of Noonan syndrome, a common autosomal disorder, especially for atypical patients[47]. Moreover, by applying AI deep learning to set up a combined model of multiple clinical tests like lung function test, bronchial experiments, and some biochemical tests from 556 patients, the prediction and diagnosis of initial asthma were remarkably promoted[48].

However, there are also scholars who hold the opposite opinion that AI has a place in clinical diagnosis, but the real influence of it may occur gradually over a long period and will not replace clinicians in the short term[49]. Overall, at the current stage, AI plus diagnosis has been the general trend and will continue to flourish.

## 2 AI IN MEDICAL TREATMENT

### 2.1 AI in Surgery

In the surgical field, the most outstanding achievement and application of AI could not be without the surgical AI system. About 20 years ago, PUMA-560, Probot, AESOP, Robodoc, and Acrobot[50–52] played an favorable auxiliary role in surgery. But in the early phase, all of the surgical systems could not operate without human control, meaning that the systems were just another kind of unintelligent scalpel that was more flexible.

With the development of AI technology, the conception of an AI plus surgical system had been raised. In the contemporary era, the most groundbreaking production of this conception is the Da Vinci surgical AI system. As a great invention unprecedented in human history, the emergence of the Da Vinci surgical system makes surgical treatment more minimally invasive, with the advantages of a clearer image, more accurate and convenient operation, and even remote operation. This creative invention allows complex surgical operations to be performed through minimally invasive methods that have been difficult in the past. There are three components in the Da Vinci surgical AI system: surgeon console, manipulator operating system, and imaging system. In 2000, the U.S. Food and Drug Administration approved the application of the Da Vinci surgical system in clinical surgery. This AI system revolutionized the traditional surgical model. For example, with the application of the Da Vinci surgical AI system, thyroid surgery was improved in terms of the postoperative cosmesis and voice outcomes[53], maxillary surgery was improved in terms of the accuracy and safety[54], gastric, nephritic, and prostatic surgery was improved as shown by the high surgical success rate but low complication rate[55–57], and lung cancer surgery was beneficial to the patients in terms of the postoperative recovery[58]. Except for the above points of surgical operation enhancement, compared to traditional surgical systems, the most outstanding characteristic of AI surgical systems particularly lies in "AI", meaning that the surgical systems progressed from a nonintelligent form to an intelligent form. Profited by the algorithms like deep learning of AI technology, the histological diagnosis *in vivo* and *in situ* during surgery stands on the stage of pathology, making efficient incisal edge pathological analysis and real-time tissue biopsy a reality[59]. By using deep learning, the AI algorithm can also self-deduct based on the abundant experiments from clinical surgeons and reconstruct clinical digitized data by uploading the surgical program to an AI surgical system to assist the surgery intelligently, including surgical excision range formulation, postoperative organ residual volume insurance, and prediction of lymph nodes with possibly positive metastasis[60]. The surgical planning and methods not only rely on surgeons, but they also depend on the program using the intelligent algorithm[61]. Of course, in the current stage, although AI surgical systems have achieved partial intelligence, they also need human supervision to a certain extent. However, this point will be further developed and be a hotspot with a bright future, and one day whole intelligence will be realized.

**2.2 AI in the Perioperative Period**

The perioperative period is the period around the whole operation, from the patient receiving surgical treatment to a basic recovery; it includes three parts: preoperative preparation, surgical period, and postoperative recovery period. During the whole process of the perioperative period, there are also lots of achievements with the application of AI technologies.

**2.2.1 Three-dimensional Printing (3DP)** 3DP is a technology that partly employs AI technology during its processes. It is one type of rapid prototyping technology that uses powdered metal or other adhesive biomaterials to construct objects by layer-by-layer printing based on digital model files (created from CT or MRI data with AI technology). The clinical imaging data are imported into intelligent software like MIMICS. After artificially selecting the regions of interest by the operator, the software can conveniently output a primary virtual three-dimensional reconstruction by algorithm analysis for printing. Although further processing by humans may be necessary at the current stage, we believe that one day it will achieve whole intelligence. Once the technology was applied in medicine, it vastly promoted the development of medicine, especially in surgery. For example, during the preoperative preparation, in the face of some complex visceral injuries or bone fractures, it is difficult for clinical surgeons to recognize the key point by traditional detections in emergency circumstances. However, combined with the initial stage of 3DP technology–Model Printing, doctors can hold the 1:1 real model of the injured part reestablished from the actual CT scanning data, get more visual and intuitive information, make more detailed preoperative plans, and even practice a simulated surgery on the model in advance[62]. Scholars have indicated that in cardiac and vascular surgery, 3DP technology provides a characteristic patient-specific model that recognizes complex anatomy and is helpful for injury orientation, plan-making, and communication with patients[63]. In addition, much research has shown that 3DP plays a vital role in the preoperative preparation for dental surgery, orthopedic surgery, spine surgery, urological surgery, and some tumor surgeries, from strengthening preoperative planning to enhancing the operator's confidence for the surgery[64–67]. Not only in the preoperative preparation but also during the surgical period, 3DP still plays a crucial role in its next stage–Surgical Guide. In a surgical operation, there are always some demands of internal fixation and cutting for orthotics or tumor excision, so problems may arise, such as how to determine the appropriate angle and location of fixation for maximum effectiveness, and where the cutting edge should be to retain more normal tissue as far as possible. Through the preoperative detection data, 3DP can produce an individualized surgical guide and a template for assisting the surgery. With 3DP template navigation in spine surgery, pedicle screw insertion received a safety enhancement and was much easier than traditional methods; meanwhile, the risk of surrounding neurovascular damage was effectively reduced, and the radiation exposure was also decreased[68, 69]. Moreover, applications of 3DP template navigation in osteotomy surgeries for tibial deformities and for total knee arthroplasty have been reported; compared with conventional methods, the operation time and efficiency were significantly improved with 3DP technology[70–72]. As for tumor excision, 3DP technology helps to precisely locate and confirm the cutting edge in bone tumor surgery, providing a satisfactory postoperative result[73] and also reducing the risk of critical structure injury and saving more normal tissues[74]. What's more, the newest stage of 3DP technology–Body Implant, magically accomplishes the reconstruction of human tissue with bioactive materials including scaffold materials, functional cells, and active factors. After printing and sterilization, the implant can be applied during surgery to replace the injured and defective human tissue due to various reasons. For instance, the application of polymers, bioceramics, and composites as bioinks that print personalized bone scaffolds have been shown to enhance the surgical effect and patient satisfaction; among those receiving large mandibular defect reconstruction surgery, the defective tissue was fully repaired through 3DP technology[75]. In orthopedic surgery, combined with the mirror-replication technique, 3DP technology also brings hope to the intractable challenge of cranial defects and limb bone defects, which used to rely on bone transplantation (a surgery with lots of complications) to reestablish bone using traditional surgical methods[76–78]. Similarly, 3DP substitution also has been widely applied in the fields of neurodegenerative disease, skeletal muscle reestablishment, arthroplasty, aortic valve replacement, and pelvic diseases in urology and gynecology[79–83]. Furthermore, in the near future, 3DP technology will accomplish the goal of printing whole functional-living organs, for the next stage–Organ Bioengineering[84].

and mixed reality (MR) technologies are new types of digital holographic image technologies that are similar to 3DP in that they partly employ AI technology to reconstruct clinical data during their processes. VR is a pure virtual digital image generated by an intelligent computer algorithm that can provide some opportunities for surgeons to practice using a virtual system, without any serious consequences of operation failure, thus improving their surgical abilities[85, 86]. However, due to the lack of real-world experience, VR cannot be applied in real surgery[87]. AR is a composition of intelligent augmented information and the real environment, and, in general, it is different from VR according to its specialty in the real world. After conversion of patient data and virtual reestablishment of the critical area, with the addition of the virtual image to the real visual world, AR technology can validly assist surgery preoperatively or intraoperatively by recognizing the complex anatomical structures and navigation during the operation[88, 89]. However, due to unwieldy equipment for AR navigation systems, there are still limitations during surgery[90]. With the appearance of the newest digital holographic imaging technology MR, the combination of VR and MR breaks the boundary between virtuality and reality, effectively solving the problem. There are three features in MR: closed combination among virtuality and reality, real-time interaction, and precise matching[91]. In the MR system, which is comprised of comparably portable equipment (e.g., a wearable MR device, the Hololens, and the latest Microsoft technological production[92]), a real-time interactive location, and vivid visual experiences, the clinical surgeon can immerse themselves in the mixed surgical world and formulate a better therapeutic schedule; in addition, the doctor-patient communication is improved[93]. Due to these advantages, this new technology has of course been applied in a variety of fields, such as spine, orthopedic, liver, kidney, and skull surgeries, for intraoperative guidance assistance, thus shortening the operation time and promoting the accuracy and safety of surgery[94–98]. Additionally, MR has even met the requirements of telemedicine, which is essential for health care in rural and remote areas, as information can be shared in real time using online chatting platforms[99]. Compared with the 3DP technology mentioned above, except the more accurate navigation, MR still has some advantages in timeliness due to the fact that 3DP production may take several hours for printing[90]. What's more, MR technology also can be used to assist with postoperative rehabilitation and routine training[100, 101].

**2.2.3 Anesthesiology Assistance** AI technology also has been widely applied in anesthesiology during the perioperative period. Anesthesia is an important part of the surgical procedure that helps provide a smooth operation; however, there are lots of risks and complications during anesthesia. Combined with the application of AI technology, six aspects have been mostly promoted and have received extensive attention: (1) anesthesia depth monitoring; (2) anesthesia control; (3) adverse event prediction; (4) ultrasound assistance; (5) pain control; and (6) operating room management[102]. AI technology increases the safety of monitoring, delivery, and postoperative management, thus bringing promising developments for anesthesiology[103, 104].

**2.2.4 Rehabilitation Assistance** In the field of postoperative rehabilitation, AI technology also plays a crucial role in the process of recovery. For instance, in the intensive care unit (ICU), the application of AI wireless sensors can effectively collect patient information, reduce false alarms, and relieve challenges in the ICU[105]. With the gradual diversification of AI technology, there have been many new tools (monitoring and remote management) in the field of nursing[106]. The AI-based medical devices can be helpful during patient recovery, meeting the requirements of rehabilitation and expediting the proceedings[107]. In addition, the application of AI robots also has accelerated limb rehabilitation in complex anthropopathic action guidance and helped patients to obtain a better degree of recovery[108, 109]. What's more, AI technology also has been used to track progression and to monitor health, which may be beneficial for the management of discharged patients[110, 111].

## 3 AI IN DRUG PRODUCTION

In the traditional model, the production of drugs requires a long period, including functional target studies, drug ingredient design studies, performance tests, clinical trials, testing, and promotion; thus, even after a long period of research, new drugs may not necessarily work as well as expected. However, with the development of AI in recent years, the new technology has changed the traditional drug industry in healthcare and has facilitated new drug discovery and assembly[112, 113]. Moreover, following the gradual maturity of AI-generation drugs, both the novelty and quality of drugs have reached new heights[114]. For instance, the combination of AI prediction models and vaccine design has efficiently accelerated clinical trial processes and cut down the research and development costs and time period[115]. Deep learning technology-guided drug discovery can target proteins as designed, which would once be impossible to achieve[116]. Benefited by the strong logical deduction and automatic learning abilities of AI technology, the design and production of cancer drugs were profoundly optimized with a better therapeutic performance[117]. Additionally, the research of AI-assisted bioinformatics tools and methods also has provided a bright future for small molecule drug therapy[118]. What's more, the

3DP technology mentioned above also has brought enormous development in drug production. 3DP in drug production possesses patient-customized characteristics as well as accomplishes the selection of drug size, shape, and combination of different pharmaceutical ingredients, which may be more convenient for clinical applications[119]. Through 3DP technology, even the layers and percentage parameters in tablet coating, therapeutic release rates and patterns can also be predesigned, thus providing better curative effects[120, 121].

## 4 AI IN MEDICAL MANAGEMENT AND EDUCATION

In the traditional model, the medical management in hospital relies on the overall planning of the hospital administrative department, and there always are some management omissions and disadvantages like the unreasonable distribution of medical resources. With AI technology regulation, procedures have changed quite a bit. Some scholars have used the long-short-term memory neural network AI technology to build a prediction model and to analyze the database of patient hospital-stay time, successfully accomplishing the prediction of accurate waiting times in the emergency department of their hospital, which effectively enhanced the medical efficiency and patients' subjective experience and promoted medical resource redistribution[122]. Furthermore, research that analyzed the data of patient hospital-stay time, the route to hospital, as well as climatic and temporal elements with 10 AI algorithms was used to reduce the average hospitalization time by 7%, select the optimum number of hospital beds, and optimize hospital resources and required inputs[123].

In addition, a real-time-prediction model based on artificial neuron networks comparatively and accurately predicted the readmission rate, thus helping to make preparations for patients and improving hospital management[124]. In short, AI technology has facilitated patient counseling, hospital management, medical resource allocation, and ultimately individualized clinical care[125].

The education of medical students is the future and hope in medical developments; however, due to the massive and intricate professional knowledge required, the training period of medical students is long and difficult. The development of medical students will be hindered if they only study medical books and specimens. With the diversified application of AI technology, the learning pattern of medical students has been richer and more colorful. AI-based problem-based learning has improved student's learning and understanding, thus enhancing their knowledge of clinical diseases[126]. The combination of studying surgery with an AI system also has provided promising results with better performance and confidence of the medical students[127]. Moreover, the AI simulation-based surgical training system that combines AI and simulation together for studying surgical techniques has created a new educational tool with objective feedback, which is beneficial for student learning[128]. Not only for assistance with learning but also for supervising, AI technology had availed the monitoring of mental health and study performance of students, thus enabling universities to know the conditions of their students in a timelier fashion[129]. What's more, the 3DP and MR technology mentioned above can also provide medical students with more vivid learning opportunities, which are unavailable in conventional textbook reading. With the assistance of intelligent algorithms, three-dimensional reconstruction is different from two-dimensional books; therefore, students can utilize the 3DP medical model for studying three-dimensional anatomical structures and even practice operations on the model to improve their surgical skills[130, 131]. Furthermore, the MR technology can also help students understand the human anatomy in a more intuitive manner with any size or layer that they control, thus providing simulative surgery training with no risks[132]. Currently, 3DP or MR-based assistance methods have been widely applied in medical education.

## 5 AI IN CORONAVIRUS RESEARCH

At the end of 2019, the new coronavirus disease 2019 (COVID-19) outbreak brought threats to the world. Human health, safety, human civilization development, and the global economy were severely affected, and countless people died in the global disaster. Fortunately, huge achievements in the prophylaxis and treatment of COVID-19 were made by relying on several advanced medical methods and highly developed technologies, including AI technology. In the race to control the spread of COVID-19, AI was used to work as human intelligence in order to address the following: early detection and diagnosis, treatment monitoring, contact tracing, prediction of cases and mortality, development of drugs and vaccines, medical workload reduction, and disease prevention[133]. During the fight against COVID-19, due to the fact that the quantification and localization of pulmonary lung CT data cannot be accurately and efficiently evaluated, Zhang developed a new system based on deep learning to analyze the CT data of patients and concluded that the right lower lobe of the lung is the high occurrence area of COVID-19 pneumonia[134]. In addition, Aikaterini applied an AI machine learning algorithm in the analysis of COVID-19 CT scans, and their findings indicated that the algorithm could promote earlier detection and medical care[135]. Moreover, Tivani proposed point-of-care diagnostic services that blended radiology, pathology, and artificial intelligence all together, further assisting the diagnosis of COVID-19[136]. Additionally, Sweta performed a quick intelligent screening for potential drugs to treat COVID-19 with a drug-repositioning method; this group was able to quickly detect drugs that may be useful by using both AI- and pharmacology-based methods, thus demonstrating that this method could be helpful for COVID-19 drug design and research[137]. This method also has been confirmed by other scholars, who established a platform based on AI learning and prediction models to identify the drugs on the market with a possibility for treating COVID-19; as a result, they found more than 80 drugs with great potential[138]. Furthermore, there has been much research on AI algorithm assistance, which pushed the quick development of COVID-19 vaccines[139–141].

In general, with suitable AI-based technology, the process of early warning, diagnosis, drug research, and medical control during the fight against COVID-19 will be effectively guaranteed[142], and the pandemic will be overcome in the near future.

## 6 FUTURE OF AI

AI technology is a high-tech production that adapts to the development of the contemporary era; therefore, it is the inevitable result of the advancement of science and technology and follows the trend of time. There have been two industrial revolutions in human society, steam revolution and electrical revolution, which both profoundly changed the way of human life and promoted human civilization. Now the scientific and technical revolution, including AI technology, has already shown an irresistible trend that has grown vigorously like a raging fire. In the medical field, with the assistance of new AI technologies, the traditional medical environment has changed a lot, and patient diagnosis using radiological, pathological, endoscopic, ultrasonographic, and biochemical examinations were effectively promoted with a higher accuracy and a lower human workload. The medical treatments during the perioperative period, including the preoperative preparation, surgical period, and postoperative recovery period, were significantly enhanced with better surgical effects. In addition, AI technology also has played a crucial role in medical drug production, medical management, and medical education, taking them into a new direction. The future of AI has come, and we believe that the new revolution will be swift like the wind and bring our medical field into an unprecedented new era.

**Open Access**

This article is licensed under a Creative Commons Attribution 4.0 International License https://creativecommons.org/licenses/by/4.0/), which permits use, sharing, adaptation, distribution and reproduction in any medium or format, as long as you give appropriate credit to the original author(s) and the source, provide a link to the Creative Commons licence, and indicate if changes were made. The images or other third party material in this article are included in the article's Creative Commons licence, unless indicated otherwise in a credit line to the material. If material is not included in the article's Creative Commons licence and your intended use is not permitted by statutory regulation or exceeds the permitted use, you will need to obtain permission directly from the copyright holder. To view a copy of this licence, visit http://creativecommons.org/licenses/by/4.0/.

**Conflict of Interest Statement**

The authors declare that they have no conflicts of interest.

## References

[1] Mintz Y, Brodie R (2019). Introduction to artificial intelligence in medicine. Minim Invasive Ther Allied Technol.

[2] Kaul V, Enslin S, Gross SA (2020). The history of artificial intelligence in medicine. Gastrointest Endosc.

[3] Miller RA, Pople HJ, Myers JD (1982). Internist-1, an experimental computer-based diagnostic consultant for general internal medicine. N Engl J Med.

[4] Shortliffe EH, Davis R, Axline SG (1975). Computer-based consultations in clinical therapeutics: explanation and rule acquisition capabilities of the MYCIN system. Comput Biomed Res.

[5] Weiss S, Kulikowski CA, Safir A (1978). Glaucoma consultation by computer. Comput Biol Med.

[6] Kulikowski CA (2019). Beginnings of Artificial Intelligence in Medicine (AIM): Computational Artifice Assisting Scientific Inquiry and Clinical Art — with Reflections on Present AIM Challenges. Yearb Med Inform.

[7] Patel VL, Shortliffe EH, Stefanelli M (2009). The coming of age of artificial intelligence in medicine. Artif Intell Med.

[8] Sorrentino FS, Jurman G, De NK (2020). Application of Artificial Intelligence in Targeting Retinal Diseases. Curr Drug Targets.

[9] Heydon P, Egan C, Bolter L (2021). Prospective evaluation of an artificial intelligence-enabled algorithm for automated diabetic retinopathy screening of 30 000 patients. Br J Ophthalmol.

[10] Xie Q, Liu Y, Huang H (2020). An innovative method for screening and evaluating the degree of diabetic retinopathy and drug treatment based on artificial intelligence algorithms. Pharmacol Res.

[11] Gong J, Liu JY, Sun XW (2018). Computer-aided diagnosis of lung cancer: the effect of training data sets on classification accuracy of lung nodules. Phys Med Biol.

[12] Gong J, Liu JY, Jiang YJ (2018). Fusion of quantitative imaging features and serum biomarkers to improve performance of computer-aided diagnosis scheme for lung cancer: A preliminary study. Med Phys.

[13] Rodriguez RA, Lang K, Gubern MA (2019). Stand-Alone Artificial Intelligence for Breast Cancer Detection in Mammography: Comparison With 101 Radiologists. J Natl Cancer Inst.

[14] McKinney SM, Sieniek M, Godbole V (2020). International evaluation of an AI system for breast cancer screening. Nature.

[15] Rodriguez RA, Lang K, Gubern MA (2019). Can we reduce the workload of mammographic screening by automatic identification of normal exams with artificial intelligence? A feasibility study. Eur Radiol.

[16] Stoel BC (2019). Artificial intelligence in detecting early RA. Semin Arthritis Rheum.

[17] Acs B, Rantalainen M, Hartman J (2020). Artificial intelligence as the next step towards precision pathology. J Intern Med.

[18] Allen TC (2019). Regulating Artificial Intelligence for a Successful Pathology Future. Arch Pathol Lab Med.

[19] Bera K, Schalper KA, Rimm DL (2019). Artificial intelligence in digital pathology — new tools for diagnosis and precision oncology. Nat Rev Clin Oncol.

[20] Serag A, Ion MA, Qureshi H (2019). Translational AI and Deep Learning in Diagnostic Pathology. Front Med (Lausanne).

[21] Zemouri R, Devalland C, Valmary DS (2019). Neural network: A future in pathology?. Ann Pathol.

[22] Wang S, Yang DM, Rong R (2019). Pathology Image Analysis Using Segmentation Deep Learning Algorithms. Am J Pathol.

[23] Komura D, Ishikawa S (2019). Machine learning approaches for pathologic diagnosis. Virchows Arch.

[24] Hart S N, Flotte W, Norgan AP (2019). Classification of Melanocytic Lesions in Selected and Whole-Slide Images via Convolutional Neural Networks. J Pathol Inform.

[25] Kosaraju SC, Hao J, Koh HM (2020). Deep-Hipo: Multi-scale receptive field deep learning for histopathological image analysis. Methods.

[26] Coudray N, Ocampo PS, Sakellaropoulos T (2018). Classification and mutation prediction from non-small cell lung cancer histopathology images using deep learning. Nat Med.

[27] Iizuka O, Kanavati F, Kato K (2020). Deep Learning Models for Histopathological Classification of Gastric and Colonic Epithelial Tumours. Sci Rep.

[28] Kanavati F, Toyokawa G, Momosaki S (2020). Weakly-supervised learning for lung carcinoma classification using deep learning. Sci Rep.

[29] Wang S, Yang DM, Rong R (2019). Artificial Intelligence in Lung Cancer Pathology Image Analysis. Cancers (Basel).

[30] Jiang YQ, Xiong JH, Li HY (2020). Recognizing basal cell carcinoma on smartphone-captured digital histopathology images with a deep neural network. Br J Dermatol.

[31] Bueno G, Fernandez CM, Gonzalez L (2020). Glomerulosclerosis identification in whole slide images using semantic segmentation. Comput Methods Programs Biomed.

[32] Namikawa K, Hirasawa T, Yoshio T, *et al.* Utilizing artificial intelligence in endoscopy: a clinician’s guide. Expert Rev Gastroenterol Hepatol, 2020:1–18 10.1080/17474124.2020.1779058 32500760

[33] Gulati S, Emmanuel A, Patel M (2020). Artificial intelligence in luminal endoscopy. Ther Adv Gastrointest Endosc.

[34] Hwang Y, Lee HH, Park C (2020). An Improved Classification and Localization Approach to Small Bowel Capsule Endoscopy Using Convolutional Neural Network. Dig Endosc.

[35] He YS, Su JR, Li Z (2019). Application of artificial intelligence in gastrointestinal endoscopy. J Dig Dis.

[36] Chahal D, Byrne MF (2020). A primer on artificial intelligence and its application to endoscopy. Gastrointest Endosc.

[37] Sharma P, Pante A, Gross SA (2020). Artificial intelligence in endoscopy. Gastrointest Endosc.

[38] Nguyen DT, Pham TD, Batchuluun G (2019). Artificial Intelligence-Based Thyroid Nodule Classification Using Information from Spatial and Frequency Domains. J Clin Med.

[39] Nguyen DT, Kang JK, Pham TD (2020). Ultrasound Image-Based Diagnosis of Malignant Thyroid Nodule Using Artificial Intelligence. Sensors (Basel).

[40] Sun C, Zhang Y, Chang Q (2020). Evaluation of a deep learning-based computer-aided diagnosis system for distinguishing benign from malignant thyroid nodules in ultrasound images. Med Phys.

[41] Chen J, You H, Li K (2020). A review of thyroid gland segmentation and thyroid nodule segmentation methods for medical ultrasound images. Comput Methods Programs Biomed.

[42] Fujioka T, Mori M, Kubota K (2019). Breast Ultrasound Image Synthesis using Deep Convolutional Generative Adversarial Networks. Diagnostics (Basel).

[43] Chen CH, Lee YW, Huang YS (2019). Computer-aided diagnosis of endobronchial ultrasound images using convolutional neural network. Comput Methods Programs Biomed.

[44] Noort F, Vaart CH, Grob A (2019). Deep learning enables automatic quantitative assessment of puborectalis muscle and urogenital hiatus in plane of minimal hiatal dimensions. Ultrasound Obstet Gynecol.

[45] Abelson S, Collord G, Ng S (2018). Prediction of acute myeloid leukaemia risk in healthy individuals. Nature.

[46] Sun R, Limkin EJ, Vakalopoulou M (2018). A radiomics approach to assess tumour-infiltrating CD8 cells and response to anti-PD-1 or anti-PD-L1 immunotherapy: an imaging biomarker, retrospective multicohort study. Lancet Oncol.

[47] Li X, Yao R, Tan X (2019). Molecular and phenotypic spectrum of Noonan syndrome in Chinese patients. Clin Genet.

[48] Tomita K, Nagao R, Touge H (2019). Deep learning facilitates the diagnosis of adult asthma. Allergol Int.

[49] Demircioglu A (2019). Radiomics-AI-based image analysis. Pathologe.

[50] Jakopec M, Harris SJ, Rodriguez YB (2001). The first clinical application of a “hands-on” robotic knee surgery system. Comput Aided Surg.

[51] Cowley G (1992). Introducing “Robodoc”. A robot finds his calling—in the operating room. Newsweek.

[52] Stefano GB (2017). Robotic Surgery: Fast Forward to Telemedicine. Med Sci Monit.

[53] Tae K (2020). Robotic thyroid surgery. Auris Nasus Larynx.

[54] Stefanelli LV, Mandelaris GA, Franchina A (2020). Accuracy Evaluation of 14 Maxillary Full Arch Implant Treatments Performed with Da Vinci Bridge: A Case Series. Materials (Basel).

[55] Lenfant L, Wilson CA, Sawczyn G (2020). Single-Port Robot-Assisted Dismembered Pyeloplasty With Mini-Pfannenstiel or Peri-Umbilical Access: Initial Experience in a Single Center. Urology.

[56] Winder A, Strauss DC, Jones RL, *et al.* Robotic surgery for gastric gastrointestinal stromal tumors: A single center case series. J Surg Oncol, 2020, doi: 10.1002/jso.26053. Online ahead of print 10.1002/jso.26053 32488872

[57] Jones R, Dobbs RW, Halgrimson WR (2020). Single port robotic radical prostatectomy with the da Vinci SP platform: a step by step approach. Can J Urol.

[58] Wang Y, Meng D, Sun X (2020). A Prospective Study of Da Vinci Surgical Robotic System with Chest Wall External Nursing Interventions. Chin J Lung Cancer (Chinese).

[59] Zuo S, Yang GZ (2017). Endomicroscopy for Computer and Robot Assisted Intervention. IEEE Rev Biomed Eng.

[60] Navarrete AJ, Hashimoto DA (2020). Current applications of artificial intelligence for intraoperative decision support in surgery. Front Med.

[61] Samareh A, Chang X, Lober WB (2019). Artificial Intelligence Methods for Surgical Site Infection: Impacts on Detection, Monitoring, and Decision Making. Surg Infect (Larchmt).

[62] Tejo OA, Buj CI, Fenollosa AF (2020). 3D Printing in Medicine for Preoperative Surgical Planning: A Review. Ann Biomed Eng.

[63] Wang C, Zhang L, Qin T (2020). 3D printing in adult cardiovascular surgery and interventions: a systematic review. J Thorac Dis.

[64] Nikoyan L, Patel R (2020). Intraoral Scanner, Three-Dimensional Imaging, and Three-Dimensional Printing in the Dental Office. Dent Clin North Am.

[65] Skelley NW, Smith MJ, Ma R (2019). Three-dimensional Printing Technology in Orthopaedics. J Am Acad Orthop Surg.

[66] Yamaguchi JT, Hsu WK (2019). Three-Dimensional Printing in Minimally Invasive Spine Surgery. Curr Rev Musculoskelet Med.

[67] Bangeas P, Tsioukas V, Papadopoulos V N (2019). Role of innovative 3D printing models in the management of hepatobiliary malignancies. World J Hepatol.

[68] Feng ZH, Li XB, Phan K (2018). Design of a 3D navigation template to guide the screw trajectory in spine: a step-by-step approach using Mimics and 3-Matic software. J Spine Surg.

[69] Kashyap A, Kadur S, Mishra A (2018). Cervical pedicle screw guiding jig, an innovative solution. J Clin Orthop Trauma.

[70] Corona PS, Vicente M, Tetsworth K (2018). Preliminary results using patient-specific 3d printed models to improve preoperative planning for correction of post-traumatic tibial deformities with circular frames. Injury.

[71] Sun ML, Zhang Y, Peng Y (2020). Accuracy of a Novel 3D-Printed Patient-Specific Intramedullary Guide to Control Femoral Component Rotation in Total Knee Arthroplasty. Orthop Surg.

[72] Zhou F, Xue F, Zhang S (2020). The application of 3D printing patient specific instrumentation model in total knee arthroplasty. Saudi J Biol Sci.

[73] Park JW, Kang HG, Kim JH (2021). The application of 3D-printing technology in pelvic bone tumor surgery. J Orthop Sci.

[74] Gomez JM, Estades FJ, Meschian CS (2019). Internal Hemipelvectomy and Reconstruction Assisted by 3D Printing Technology Using Premade Intraoperative Cutting and Placement Guides in a Patient With Pelvic Sarcoma: A Case Report. JBJS Case Connect.

[75] Salah M, Tayebi L, Moharamzadeh K (2020). Three-dimensional bio-printing and bone tissue engineering: technical innovations and potential applications in maxillofacial reconstructive surgery. Maxillofac Plast Reconstr Surg.

[76] Alkhaibary A, Alharbi A, Alnefaie N (2020). Cranioplasty: A Comprehensive Review of the History, Materials, Surgical Aspects, and Complications. World Neurosurg.

[77] Vidal L, Kampleitner C, Brennan MA (2020). Reconstruction of Large Skeletal Defects: Current Clinical Therapeutic Strategies and Future Directions Using 3D Printing. Front Bioeng Biotechnol.

[78] Xing F, Xiang Z, Rommens PM (2020). 3D Bioprinting for Vascularized Tissue-Engineered Bone Fabrication. Materials (Basel).

[79] Rey F, Barzaghini B, Nardini A (2020). Advances in Tissue Engineering and Innovative Fabrication Techniques for 3-D-Structures: Translational Applications in Neurodegenerative Diseases. Cells.

[80] Boso D, Maghin E, Carraro E (2020). Extracellular Matrix-Derived Hydrogels as Biomaterial for Different Skeletal Muscle Tissue Replacements. Materials (Basel).

[81] Ettinger M, Windhagen H (2020). Individual revision arthroplasty of the knee joint. Orthopade.

[82] Levin D, Mackensen GB, Reisman M (2020). 3D Printing Applications for Transcatheter Aortic Valve Replacement. Curr Cardiol Rep.

[83] Farmer ZL, Dominguez RJ, Mancinelli C (2020). Urogynecological surgical mesh implants: New trends in materials, manufacturing and therapeutic approaches. Int J Pharm.

[84] Edgar L, Pu T, Porter B (2020). Regenerative medicine, organ bioengineering and transplantation. Br J Surg.

[85] Mirchi N, Bissonnette V, Ledwos N (2020). Artificial Neural Networks to Assess Virtual Reality Anterior Cervical Discectomy Performance. Oper Neurosurg (Hagerstown).

[86] Sadeghi AH, Taverne Y, Bogers A (2020). Immersive virtual reality surgical planning of minimally invasive coronary artery bypass for Kawasaki disease. Eur Heart J.

[87] Fertleman C, Aubugeau WP, Sher C (2018). A Discussion of Virtual Reality As a New Tool for Training Healthcare Professionals. Front Public Health.

[88] Creighton FX, Unberath M, Song T (2020). Early Feasibility Studies of Augmented Reality Navigation for Lateral Skull Base Surgery. Otol Neurotol.

[89] Gibby J, Cvetko S, Javan R (2020). Use of augmented reality for image-guided spine procedures. Eur Spine J.

[90] Hu HZ, Feng XB, Shao ZW (2019). Application and Prospect of Mixed Reality Technology in Medical Field. Curr Med Sci.

[91] Goo HW, Park SJ, Yoo SJ (2020). Advanced Medical Use of Three-Dimensional Imaging in Congenital Heart Disease: Augmented Reality, Mixed Reality, Virtual Reality, and Three-Dimensional Printing. Korean J Radiol.

[92] Salmas M, Chronopoulos E, Chytas D. Comment on: “A Novel Evaluation Model for a Mixed-Reality Surgical Navigation System: Where Microsoft HoloLens Meets the Operating Room”. Surg Innov, 2020:1611082121 10.1177/1553350620927607 32490724

[93] Wu X, Liu R, Yu J (2018). Mixed Reality Technology Launches in Orthopedic Surgery for Comprehensive Preoperative Management of Complicated Cervical Fractures. Surg Innov.

[94] Gu Y, Yao Q, Xu Y (2020). A Clinical Application Study of Mixed Reality Technology Assisted Lumbar Pedicle Screws Implantation. Med Sci Monit.

[95] Chytas D, Chronopoulos E, Salmas M (2021). Comment on: “Intraoperative 3D Hologram Support With Mixed Reality Techniques in Liver Surgery”. Ann Surg.

[96] Zeiger J, Costa A, Bederson J (2020). Use of Mixed Reality Visualization in Endoscopic Endonasal Skull Base Surgery. Oper Neurosurg (Hagerstown).

[97] Wu X, Liu R, Yu J (2018). Mixed Reality Technology-Assisted Orthopedics Surgery Navigation. Surg Innov.

[98] Yoshida S, Sugimoto M, Fukuda S (2020). Mixed reality computed tomography-based surgical planning for partial nephrectomy using a head-mounted holographic computer. Int J Urol.

[99] Rojas ME, Cabrera ME, Lin C (2020). The System for Telementoring with Augmented Reality (STAR): A head-mounted display to improve surgical coaching and confidence in remote areas. Surgery.

[100] Held J, Yu K, Pyles C (2020). Augmented Reality-Based Rehabilitation of Gait Impairments: Case Report. JMIR Mhealth Uhealth.

[101] Chen PJ, Penn IW, Wei SH (2020). Augmented reality-assisted training with selected Tai-Chi movements improves balance control and increases lower limb muscle strength in older adults: A prospective randomized trial. J Exerc Sci Fit.

[102] Hashimoto DA, Witkowski E, Gao L (2020). Artificial Intelligence in Anesthesiology: Current Techniques, Clinical Applications, and Limitations. Anesthesiology.

[103] Seger C, Cannesson M (2020). Recent advances in the technology of anesthesia. F1000Res.

[104] Kamdar N, Jalilian L (2020). Telemedicine: A Digital Interface for Perioperative Anesthetic Care. Anesth Analg.

[105] Poncette AS, Mosch L, Spies C (2020). Improvements in Patient Monitoring in the Intensive Care Unit: Survey Study. J Med Internet Res.

[106] Angehrn Z, Haldna L, Zandvliet AS (2020). Artificial Intelligence and Machine Learning Applied at the Point of Care. Front Pharmacol.

[107] Dai B, Yu Y, Huang L (2020). Application of neural network model in assisting device fitting for low vision patients. Ann Transl Med.

[108] Averta G, Della SC, Valenza G (2020). Exploiting upper-limb functional principal components for humanlike motion generation of anthropomorphic robots. J Neuroeng Rehabil.

[109] Zhao Y, Liang C, Gu Z (2020). A New Design Scheme for Intelligent Upper Limb Rehabilitation Training Robot. Int J Environ Res Public Health.

[110] De CH, Corradi F, Smeets C (2020). Wearable Monitoring and Interpretable Machine Learning Can Objectively Track Progression in Patients during Cardiac Rehabilitation. Sensors (Basel).

[111] Ramezani R, Zhang W, Xie Z (2019). A Combination of Indoor Localization and Wearable Sensor-Based Physical Activity Recognition to Assess Older Patients Undergoing Subacute Rehabilitation: Baseline Study Results. JMIR Mhealth Uhealth.

[112] Bajorath J, Kearnes S, Walters WP (2020). Artificial Intelligence in Drug Discovery: Into the Great Wide Open. J Med Chem.

[113] Brown N, Ertl P, Lewis R (2020). Artificial intelligence in chemistry and drug design. J Comput Aided Mol Des.

[114] Zhavoronkov A (2020). Medicinal Chemists versus Machines Challenge: What Will It Take to Adopt and Advance Artificial Intelligence for Drug Discovery?. J Chem Inf Model.

[115] Russo G, Reche P, Pennisi M, *et al.* The combination of artificial intelligence and systems biology for intelligent vaccine design. Expert Opin Drug Discov, 2020:1–15 10.1080/17460441.2020.1791076 32662677

[116] Fernandez A (2020). Artificial Intelligence Teaches Drugs to Target Proteins by Tackling the Induced Folding Problem. Mol Pharm.

[117] Liang G, Fan W, Luo H (2020). The emerging roles of artificial intelligence in cancer drug development and precision therapy. Biomed Pharmacother.

[118] Takakusagi Y, Takakusagi K, Sakaguchi K, *et al.* Phage display technology for target determination of small-molecule therapeutics: an update. Expert Opin Drug Discov, 2020:1–13 10.1080/17460441.2020.1790523 32660284

[119] Awad A, Fina F, Goyanes A (2020). 3D printing: Principles and pharmaceutical applications of selective laser sintering. Int J Pharm.

[120] Pandey M, Choudhury H, Fern J (2020). 3D printing for oral drug delivery: a new tool to customize drug delivery. Drug Deliv Transl Res.

[121] Tsintavi E, Rekkas DM, Bettini R (2020). Partial tablet coating by 3D printing. Int J Pharm.

[122] Cheng N, Kuo A (2020). Using Long Short-Term Memory (LSTM) Neural Networks to Predict Emergency Department Wait Time. Stud Health Technol Inform.

[123] Nas S, Koyuncu M (2019). Emergency Department Capacity Planning: A Recurrent Neural Network and Simulation Approach. Comput Math Methods Med.

[124] Saab A, Saikali M, Lamy JB (2020). Comparison of Machine Learning Algorithms for Classifying Adverse-Event Related 30-Day Hospital Readmissions: Potential Implications for Patient Safety. Stud Health Technol Inform.

[125] Lin YW, Zhou Y, Faghri F (2019). Analysis and prediction of unplanned intensive care unit readmission using recurrent neural networks with long short-term memory. PLoS One.

[126] Wu D, Xiang Y, Wu X (2020). Artificial intelligence-tutoring problem-based learning in ophthalmology clerkship. Ann Transl Med.

[127] Yang YY, Shulruf B (2019). Expert-led and artificial intelligence (AI) system-assisted tutoring course increase confidence of Chinese medical interns on suturing and ligature skills: prospective pilot study. J Educ Eval Health Prof.

[128] Mirchi N, Bissonnette V, Yilmaz R (2020). The Virtual Operative Assistant: An explainable artificial intelligence tool for simulation-based training in surgery and medicine. PLoS One.

[129] Dekker I, De Jong EM, Schippers MC (2020). Optimizing Students’ Mental Health and Academic Performance: AI-Enhanced Life Crafting. Front Psychol.

[130] Bertin H, Huon JF, Praud M (2020). Bilateral sagittal split osteotomy training on mandibular 3-dimensional printed models for maxillofacial surgical residents. Br J Oral Maxillofac Surg.

[131] Bohl MA, McBryan S, Pais D (2020). The Living Spine Model: A Biomimetic Surgical Training and Education Tool. Oper Neurosurg (Hagerstown).

[132] Sappenfield JW, Smith WB, Cooper LA (2018). Visualization Improves Supraclavicular Access to the Subclavian Vein in a Mixed Reality Simulator. Anesth Analg.

[133] Vaishya R, Javaid M, Khan IH (2020). Artificial Intelligence (AI) applications for COVID-19 pandemic. Diabetes Metab Syndr.

[134] Zhang HT, Zhang JS, Zhang HH (2020). Automated detection and quantification of COVID-19 pneumonia: CT imaging analysis by a deep learning-based software. Eur J Nucl Med Mol Imaging.

[135] Sakagianni A, Feretzakis G, Kalles D (2020). Setting up an Easy-to-Use Machine Learning Pipeline for Medical Decision Support: A Case Study for COVID-19 Diagnosis Based on Deep Learning with CT Scans. Stud Health Technol Inform.

[136] Mashamba TP, Drain PK (2020). Point-of-Care Diagnostic Services as an Integral Part of Health Services during the Novel Coronavirus 2019 Era. Diagnostics (Basel).

[137] Mohanty S, Harun AR, Mridul M (2020). Application of Artificial Intelligence in COVID-19 drug repurposing. Diabetes Metab Syndr.

[138] Ke YY, Peng TT, Yeh TK (2020). Artificial intelligence approach fighting COVID-19 with repurposing drugs. Biomed J.

[139] Kim J, Zhang J, Cha Y (2020). Advanced bioinformatics rapidly identifies existing therapeutics for patients with coronavirus disease-2019 (COVID-19). J Transl Med.

[140] Arash KA, Julia W, Milad S (2020). Artificial Intelligence for COVID-19 Drug Discovery and Vaccine Development. Front Artif Intell.

[141] Elaziz MA, Hosny KM, Salah A (2020). New machine learning method for image-based diagnosis of COVID-19. PLoS One.

[142] Mali SN, Pratap AP (2021). Targeting infectious Coronavirus Disease 2019 (COVID-19) with Artificial Intelligence (AI) applications: Evidence based opinion. Infect Disord Drug Targets.

